# A critical analysis of national plans for climate adaptation for health in South America

**DOI:** 10.1016/j.lana.2023.100604

**Published:** 2023-10-09

**Authors:** Valerie A. Paz-Soldán, Ariana Valcarcel, Katya Canal-Solis, Zaray Miranda-Chacon, Yasna K. Palmeiro-Silva, Stella M. Hartinger, Ana G. Suárez-Linares, Valeria Falla-Valdez, Claudio Intimayta-Escalante, Mariana Lehoucq, Angelica Pretell, Ricardo Castillo-Neyra

**Affiliations:** a Tulane University School of Public Health and Tropical Medicine, New Orleans, LA, USA; b Delft University of Technology, Delft, the Netherlands; c University of Leeds, School of Earth and Environment, Leeds, UK; d Anatomy Department, School of Medicine, Universidad de Costa Rica, San José, Costa Rica; e Institute for Global Health, University College London, London, UK; f School of Public Health and Management "Carlos Vidal Layseca", Universidad Peruana Cayetano Heredia, Lima, Peru; g Department of Biostatistics, Epidemiology and Informatics, Perelman School of Medicine, University of Pennsylvania, PA, USA

**Keywords:** Climate change, Climate adaptation, South America, Health, Health policy, Policy analysis, Public policy, Vulnerable populations, Global health

## Abstract

Climate adaptation measures are critical for protecting human health. National Adaptation Plans (NAPs), Nationally Determined Contributions (NDCs), and National Communications (NCs) play a crucial role in helping countries identify, analyze, and address their vulnerabilities to climate change impacts, while also assessing available resources and capacities. This study aimed to assess the comprehensiveness of South American countries' NAPs, NDCs, and NCs in addressing the effects of climate change on health. A total of 38 NAPs, NDCs, and NCs of 12 South American countries were analysed. Ad hoc scores were developed to assess baseline information, adaptation proposals, identification of involved institutions, funding needs and allocation, measurable progress indicators, and coherence. Overall, all South American countries have NDCs and NCs, and seven have NAPs. In most countries, the intersectoral health analysis revealed a lack of linkage to health issues related to that sector. Additionally, most planning documents lack detailed information to guide policymakers in taking practical actions; areas with low scores include allocation of funds, involvement of health-related institutions, and measurable indicators. While South American countries acknowledge the health impacts of climate change in their plans, enhancing public health protection requires maximizing climate policy benefits and including health-related issues across all relevant sectors.

**Funding:**

This study was not funded. However, three co-authors received funding for some of their time: AV and KC were supported by the Wellcome Trust (209734/Z/17/Z); RCN was funded by K01AI139284 (NIH-NIAID). Funding for the publication was provided by Universidad Peruana Cayetano Heredia.

## Introduction

Countries in South America (SA) collectively contribute less than 10% of global greenhouse gas (GHG) emissions.^1^ However, the population—as is the case globally—is experiencing the harmful effects of climate change.^2^ An increasing proportion of people are exposed to heat waves, droughts, wildfires, vector-borne diseases, and other hazards.^2^, ^3^, ^4^ These threats are exacerbated by high levels of poverty, gender inequality, limited access to basic infrastructure services, and state fragility; all of these characteristics of systemic vulnerability, and factors that are necessary to understand the societal impacts of climate change.^5^, ^6^, ^7^ As a result, the health and well-being of the populations are significantly impacted.^2^^,^^8^ An immediate and integrated response to climate change is imperative at all levels of governance and across the entire region to address these challenges effectively.

Mitigation efforts to reduce atmospheric GHGs are not only necessary due to the collective global responsibility to stop anthropogenic climate change, but also because of the immense local and immediate benefits of GHG emission reduction, such as cleaner air.^2^ However, we have reached a critical juncture, where even substantial reductions in global and regional emissions would not immediately eliminate the long-lasting effects of climate change that may well persist for decades. These effects will disproportionately impact the most vulnerable individuals, including indigenous people, the elderly, people with pre-existing health conditions, outdoor agriculture workers, and children.^9^ Climate adaptation measures are essential to safeguard the health and wellbeing of populations in South America.

Climate adaptation—or the “process of adjustment to actual or expected climate and its effects, in order to moderate harm or exploit beneficial opportunities”^8^—requires that governments and communities iteratively analyse their social contexts, identify key hazards and vulnerabilities, and assess available resources to promote tailored and effective climate adaptation strategies. This approach is supported by international frameworks such as the Cancun Adaptation Framework^10^ and the Paris Agreement,^11^ which emphasise the development of National Adaptation Plans (NAPs), Nationally Determined Contributions (NDCs), and National Communications (NCs) to facilitate comprehensive planning and coordinated actions for adaptation at the national level.

NAPs play a crucial role in helping countries identify, analyse, and address their current and future vulnerabilities to climate change impacts, while also assessing available resources and capacities. NAPs are designed to lay out a long-term and iterative process that facilitates the development and implementation of climate adaptation strategies and programmes. Globally, a minority of low- or middle-income countries have adopted umbrella policies and others include sector-specific NAPs with targeted goals, such as addressing health impacts.^12^ Complementarily, NDCs reflect countries’ commitments and targets to reduce national GHG emissions and strengthen climate adaptation processes. NDCs outline emissions reduction targets and specific policies and measures to achieve those targets. They may also outline a country's adaptation plans and identify financial, technological, or capacity-building support required for implementation.^13^ Finally, NCs enable countries to report on their actions to mitigate GHG emissions and adapt to the impacts of climate change. NCs serve as critical tools for monitoring and evaluating progress towards the goals of the Paris Agreement.^14^

Considering the complexity associated with climate adaptation, it is crucial that countries develop integrated climate planning documents—NAPs, NDCs, and NCs—that align with broader social and health programs and agendas. This approach requires robust intersectoral and multilevel coordination to enable countries to capitalise on the multiple benefits of public policies and optimise resource utilisation. The responsibility for promoting health and wellbeing among populations does not solely rest with the health sector, but also involves other sectors that strongly impact the health and well-being of populations (hereafter referred to as health-impacting sectors), such as water resources, disaster risk management, agriculture, among others.

The objective of the Lancet Countdown South America working group on adaptation, planning, and resilience for health was to evaluate the scope and depth of South American sovereign countries’ NAPs, NDCs, and NCs (hereinafter referred to as plans or planning documents) regarding the impact of climate change on health outcomes. There were two main analyses. First, the research team evaluated to what extent health-related topics or measures were included in the plans of health-impacting sectors (an intersectoral health analysis). Second, the team analysed climate-sensitive health topics, such as malnutrition, infectious diseases, and disaster risks, that were included within the health sector plans or in the health sections of the plans (a health-sector-focused analysis). This assessment provided insights into the extent to which these plans address the health risks associated with climate change. These two complementary analyses were designed to identify areas of strength and weakness in the current plans, providing recommendations for future versions of the planning documents. This work contributes to a growing body of knowledge on climate change and health policies in South America, supporting efforts to align adaptation strategies with health outcomes.

## Search strategy and analysis

### Design and procedures

A critical analysis of planning documents, specifically NAPs, NDCs, and NCs, was conducted for this study. These documents were selected because they constitute key policy instruments at international and national levels and serve as national umbrella policies to guide adaptation measures. NAPs, NDCs, and NCs per country were analysed as one body of national policy documentation since their individual purposes varied by country and not all South American countries had the three documents.

First, to ensure a systematic data retrieval process, plans were identified and retrieved from the UNFCCC repositories and complementary sources (see data sources and collection section). Second, after numerous discussions about potential conceptual frameworks that could be used to organise the analytical process of different types and scopes of documents, including the Sustainable Livelihoods Approach^15^ and the One Health Approach,^16^ the research team recognized the need to develop two sets of scoring cards or rubrics with analytical criteria for the various topics covered in documents, as well as cross-cutting themes (i.e., indicators, funding allocation) (see Data analysis section). These scoring cards were developed ad hoc by the research team relying on international standards in health and climatic adaptation policies.^12^^,^^17^^,^^18^

The first scoring card examined the inclusion of health issues or health-related activities in sectors which are considered to have significant impacts on the social determinants of health (hereon “intersectoral health analysis”).^19^ For this intersectoral health analysis, the health-impacting sectors included: i) agriculture and food production; ii) biodiversity, environment, and ecosystems; iii) cities and infrastructure; iv) education and communication; v) disasters risk management; vi) energy; vii) health; viii) industry; ix) tourism; and x) water resources. Each of these sectors are usually included as chapters or sections in the plans; however, some plans include them as cross-cutting sectors or themes.

The second scoring card analysed climate-sensitive health topics in the health sections of the plans or within health sector plans (hereon “health-sector-focused analysis”). Based on the scientific evidence, seven topics are identified as health or health-related impacts of climate change: i) infectious diseases, ii) cardiorespiratory diseases, iii) malnutrition, iv) injuries, v) mental health disorders, vi) health infrastructure, and vii) disaster risks.^8^^,^^20^ These topics currently represent a significant burden of disease in South America^21^ and are highly influenced by changes in the climate and ecosystems; hence, analyses of projections are rather relevant for policymaking.

The third step involved analysing and scoring manually the information gathered from the planning documents. Two reviewers independently examined each plan using the two scoring cards specifically developed for these analyses. A third reviewer met with the two reviewers to discuss and resolve any discrepancies. This rigorous process ensured a systemic analysis of these climate adaptation plans.

### Data sources and collection

A total of 38 national climate change planning documents were reviewed and scored,^22^, ^23^, ^24^, ^25^, ^26^, ^27^, ^28^, ^29^, ^30^, ^31^, ^32^, ^33^, ^34^, ^35^, ^36^, ^37^, ^38^, ^39^, ^40^, ^41^, ^42^, ^43^, ^44^, ^45^, ^46^, ^47^, ^48^, ^49^, ^50^, ^51^, ^52^, ^53^, ^54^, ^55^, ^56^, ^57^, ^58^, ^59^ including NAPs, sectoral NAPs, NDCs, and NCs from 12 sovereign countries of South America (Argentina, Bolivia, Brazil, Chile, Colombia, Ecuador, Guyana, Paraguay, Peru, Suriname, Uruguay, and Venezuela). Non-sovereign South American countries (e.g., French Guiana) or other territories were not included in the analysis as the actual governments responsible for formulating policies are outside of the South American geographical boundaries. The plans were retrieved from the United Nations Framework Convention on Climate Change (UNFCCC) website (www.unfccc.int) in December 2021. As countries submit these plans in different dates, the latest plans (up to 2021) submitted to UNFCCC were retrieved, included, and analysed. Supplementary Table S1 in the Supplementary Material shows all plans included in this study.

### Data extraction

Given the diverse structures and contents of each country's plan, standardized criteria were established for the manual extraction and analysis of the data.

All documents were assigned to two reviewers who independently read, created notes on each item which included document page numbers and exact quotes, and provided a tentative score on a rubric sheet created on Excel. The two reviewers independently looked for information on health, health linkages, or health-related activities for the intersectoral health analysis, as well as for information on climate-sensitive health topics in their assigned plans. They took notes and cut and paste sections into a virtual whiteboard to ensure information was consolidated for the scoring process, as well as for the discussion with the third reviewer when scoring discrepancies emerged.

The scoring cards for both the intersectoral health analysis and the health-sector-focused analysis coded information as “explicit” if the health topic was clearly described in the document, or “implicit” if only implied (Table 1). Explicit information for the health-sector-focused analysis contained the keywords, concepts, or synonyms: “infectious diseases”, “cardiorespiratory diseases”, “malnutrition”, “injuries”, “mental health disorders”, “health infrastructure”, and “risk disaster management”. In contrast, implicit information for both analyses reflected the topics in a broad manner (see Supplementary Tables S2 and S3 in the Supplementary Material).

**Table 1:** Criteria for analysing intersectoral inclusion of health issues or activities in health-impacting sectors.

Score	Baseline: *Is there information or existing research (context) about the topic, or is the health problem or outcome clearly defined?*	Adaptation proposals: *Are there any suggestions, plans, or actions defined to address the described problems?*	Leading institution and involvement: *Does the plan define the key institution(s) involved, including the one taking the lead?*	Financing: *Is there any information of a global or specific amount needed or who it will be obtained from, and is this detailed, or is it a generic mention?*	Indicators: *Does the plan describe indicators or how will they measure the fulfilment of their objectives or proposals?*
0	There is superficial or no mention of the subject related to health	There are no proposals of the subject as it relates to health	Health sector is not involved	There is no mention about it	No indicators
1	Includes context with an implicit relation to health	Has a broad proposal with an implicit relation to health	Health sector or related institutions are mentioned	General amount needed by prioritised sector	Has more than two indicators with an implicit relation to health
2	Recognises problems with an explicit or implicit relation to health	Mentions specific actions with an explicit or implicit relation to health	Health sector or related institutions are involved in proposals, actions, or indicators	Detailed amounts needed and/or obtained for more than one action that involves health	Has more than two indicators with an explicit relation to health
3	Has a thorough analysis of the problem(s) and an explicit relation to health	Detailed actions and how to achieve the objectives with an explicit relation to health	Health sector or related institutions are involved in proposals, actions, or indicators, and have detailed responsibilities or goals with deadlines	Detailed amounts needed and/or obtained for more than one action that involves health and potential funding sources and/or budgets already obtained	Has more than two indicators and describes how these will be monitored, with an explicit relation to health

### Data analysis

Key information was extracted from the various planning documents and organised systematically into an electronic scoring card in excel for the analysis. To ensure rigour in the scoring process, detailed notes with direct quotes and references to documents and page numbers were used during the meeting that took place when both reviewers were done reviewing all documents for a country.

To enhance consistency and minimise bias in the coding process, two reviewers independently examined each plan using the coding definitions of the two scoring cards specifically developed for these analyses. A third reviewer met with the two reviewers to discuss and resolve any coding discrepancies. Specifically, when both reviewers were done reviewing all documents for a country, a meeting was set with a third researcher; each item on the scoring card was discussed thoroughly, with the two individuals referring to specific quotes and page numbers of documents to provide examples of what they found. Finalising the discussion on each scored item, the three individuals discussed a final score for each item and registered this number on a virtual board shared by the team, before moving on to the next discussion. Due to the magnitude of data processed and referred to by our reviewers per item, and our focus on a panoramic assessment of scope and depth of planning documents, we do not provide all the culled information in this manuscript (see Supplementary Table S27 in Supplemental Materials to view an example of detailed policies for some criteria). This process allowed for verification of findings in the data extraction process and ensured the accuracy and reliability of the findings for a rigorous systemic analysis of these climate adaptation plans.

Initially, we formulated a binary score (0 and 1) for the intersectoral and health-sector-focused analyses. However, early on, we realised that most sectors in most country plans did not have explicit information about linkages with health; therefore, for the intersectoral analysis, we developed a 4-level scoring system to capture the gradient of implicit-explicit linkage to health (Table 1). For the health-sector-focused analysis, we kept the binary score (Table 2) since explicit linkages with health were expected and found in this sector.

**Table 2:** Criteria for analysing the inclusion of climate-sensitive health topics in health plans.

Score	Baseline: *Is there information or existing research (context) about the topic, or is the health problem or outcome clearly defined?*	Adaptation proposals: *Are there any suggestions, plans, or actions defined to address the described problems?*	Financing: *Is there any information of a general or specific amount needed or obtained, and is this detailed or generic?*	Indicators: *Does the plan describe indicators or how will they measure the fulfilment of their objectives or proposals?*	Coherence: *Is there coherence between the detected or identified problems, with the adaptation proposals or plans and/or indicators?*
0	There is a limited or no mention of the subject	There are no proposals, actions, nor suggestions	There is no mention about funding needed	There are no indicators	There is no coherence between baseline, adaptation proposals and/or indicators
1	There are a minimum of two detected problems with context included	There are a minimum of two proposals, actions, and/or suggestions	There is mention of at least a general amount of funding needed	There are a minimum of two indicators	There is coherence between baseline, adaptation proposals, and/or indicators

Not all plans follow the same structure; some plans include health-impacting sectors as cross-cutting sectors or themes (e.g., risks and disasters management) whereas other countries had a specific health sector. Each criterion was assigned a score on a scale from 0 to 3, with 0 representing limited or no linkage to health, and 3 representing detailed linkage to health.

### Intersectoral health analysis

A scoring card for the intersectoral health analysis with five criteria was developed. The five criteria evaluated included: i) baseline information available for that health issue, ii) adaptation proposals for that issue, iii) identification of leading institution and/or different institutions to lead the planning and implementation of any proposal related to this health issue, iv) specifics about funding needed or who will finance this issue, and v) indicators have been identified for measuring this issue/topic (Table 1). Not all plans follow the same structure; some plans include health-impacting sectors as cross-cutting sectors or themes (e.g., risks and disasters management) whereas others had a specific health sector. Each criterion was assigned a scored on a scale from 0 to 3, with 0 representing limited or no information, and 3 representing detailed information on that topic.

### Health-sector-focused analysis

To evaluate the inclusion of key climate-sensitive health topics in health plans or sections of plans, five criteria were proposed and scored on a binary scale, with 0 for “no evidence or only mentioned of this item with no depth” and 1 for “more than one mention of the category and criteria”. The five criteria were similar to that of the intersectoral health analysis and included baseline information, adaptation proposals, indicators, financing, but instead of using the category of “leading institution and involvement”, the coders scored the coherence within the plan (Table 2).

## Descriptive analysis

### Intersectoral health analysis

**Fig. 1:**
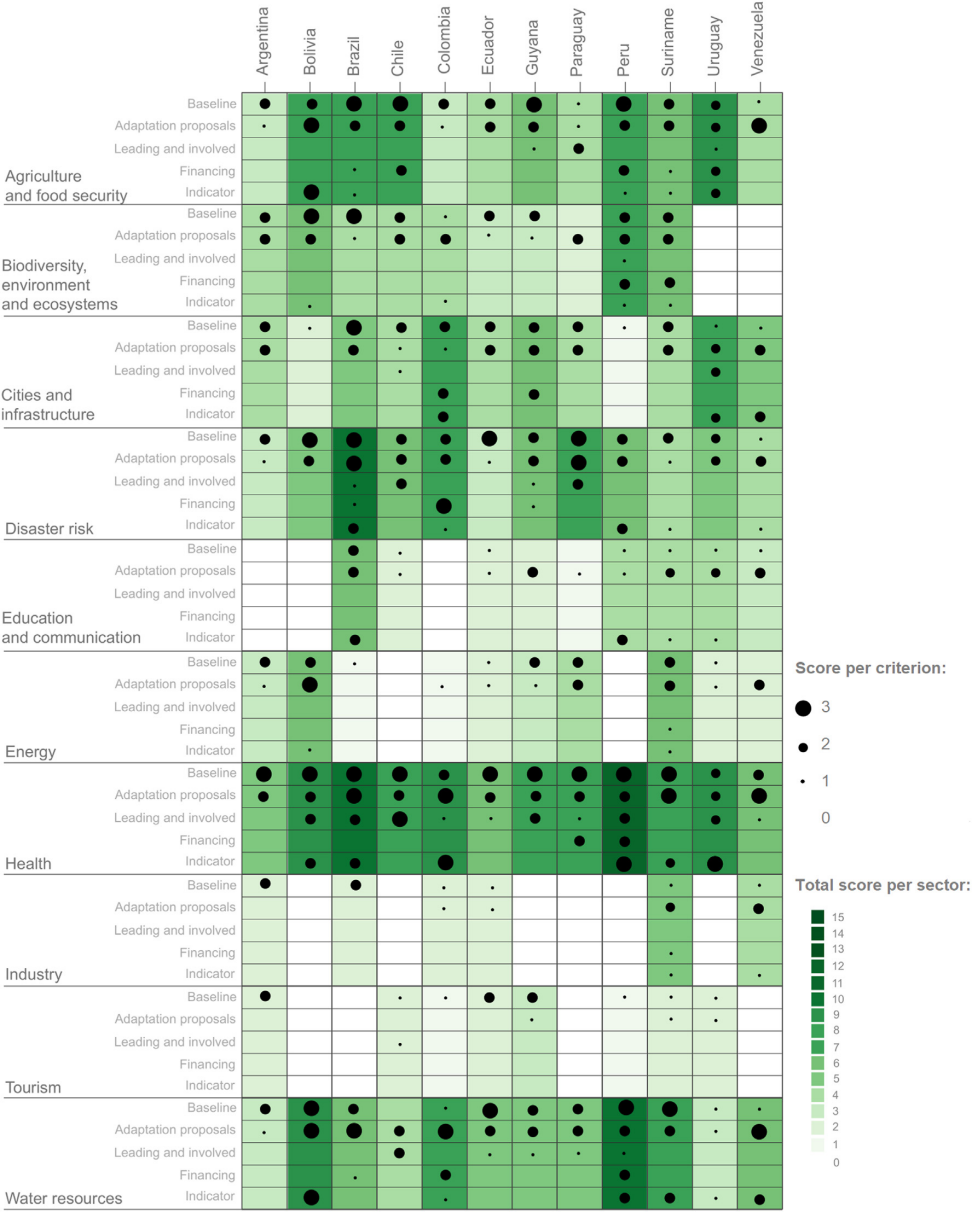
Intersectoral health analysis of plans in South American countries.

As of December 2021, all South American countries have NDCs, NCs, and seven have NAPs, with a few countries having sectoral NAPs (Brazil, Chile, and Uruguay), and one country having a health NAP (Chile) (see Supplementary Table S1 in the Supplementary Material). A total of 38 documents were reviewed in detail. All NAPs, NDCs, and NCs include health topics and recognise the health sector or health population as a vulnerable and relevant sector or area for further policies and actions. However, the depth and scope of this inclusion varied significantly by plan and by country. Some countries have specific chapters, sections, or measures dedicated to addressing the health impacts of climate change, while others briefly mention health-related activities. Fig. 1 summarises the main findings of this analysis. The figure presents the overall score represented proportionally through the spectrum of green-scale colours for each health-impacting sector and each South American country. The figure also presents the individual scores per criterion using proportionally sized black dots, where larger dots correspond to more explicit linkage to health.

The plans include detailed information on the types of impact of climate change on population health and health systems; however, the details on the planning or implementation of the adaptation measures to reduce vulnerability, as well as specific funding allocation and indicators to evaluate baseline conditions and future evaluations, are less clear and developed for all countries. Seven countries (Brazil, Chile, Colombia, Paraguay, Peru, Uruguay, and Surinam) made a distinction between urban and rural areas in their national plans regarding infrastructure. Specifically, they use this distinction to discuss the availability of resources (e.g., energy, water, housing, food) which impact health services and communities’ well-being; however, no direct connection was made regarding health. Detailing plans for urban and rural communities will be important to respond to differences in systemic vulnerabilities^7^ between these two populations.

Most plans include baseline information (highest scored criterion), focusing on impacts of climate change on vector-borne diseases, infectious and respiratory diseases, general morbidity and mortality, exposure to climate extremes, and challenges related to healthcare access. There was baseline information on climatic hazards and their geographical distribution, as well as potential impacts on population health and health systems. However, importantly, this information was predominantly based on international literature rather than focusing on national realities, local exposures, and vulnerabilities, or national level projections regarding the expected impact of climate change within the country. Baseline information that included national data on the different subject matters was limited.

Proposals on adaptation plans were the second highest scored criterion across all plans. Although these proposals are rather general and not well linked to funding, responsible lead institutions, and indicators, the proposals represent governments’ efforts to plan adaptation actions. The adaptation proposals are distributed across a few health-impacting sectors, mainly health, agriculture and food production, disaster risk management, energy, and water resources. Some examples that represent clear adaptation proposals are Peru and Paraguay: both countries’ NAPs include the importance of a monitoring and epidemiological surveillance system for different climate scenarios and explain how to achieve their objectives. Even more important is the actual implementation of these proposals: Argentina and Brazil have created their climate observatories with capacity to analyse, predict, and operationalize response.

Overall, most of the plans scored low in leading institutions, funding, and indicators (see Table 1 for definitions). With regards to leading institutions, although the Ministry of Health is generally mentioned as a leading institution, specific details about departments or units that would lead the planning or subsequent work are missing, as well as details about intersectoral or multilevel groups to carry out the work. There are exceptions: Chile’s Health NAP was very specific about the Department or Office that will direct specific adaptation plans and even the position of the person in charge of its implementation, hence detailing the agencies and individuals involved in the adaptation planning for specific items. The health-impacting sectors that frequently included health institutions as leading institutions were agriculture and food production, risks and disaster management, and water resources.

Most plans are missing information about funding for health-related adaptation plans and actions—or where this funding comes from. Only two countries indicate a specific amount needed for the health sector planning: Peru’s NAP indicates an overall financial amount required for the entire sector and, in more detail, Paraguay’s NAP specifies overall and specific amounts for infectious diseases, diarrhoeal diseases, and respiratory illnesses (see Supplementary Materials Panel S1).

Regarding measurable indicators related to health, half of the countries mention them at some point in their plans: Bolivia, Brazil, Colombia, Peru, Uruguay, and Suriname. There are particular good examples of this: Colombia in its NDC, and Peru and Suriname in their NAPs (see Colombia indicator in Supplementary Materials Panel S1). In all three cases, their goals, objectives, indicators, responsible entities, and timeline of activities are specifically described. It should be noted that Colombia's NDC even links its goals to the Sustainable Development Goals; Peru's NAP emphasises the enabling conditions, benefits, and co-benefits of the implementation of the action; and Suriname's NAP describes strategic outcomes, adaptive measures, and indicator outputs.

In terms of the health-impacting sectors that include a health perspective or health-related activities, the highest scores were obtained in agriculture and food production; biodiversity, environment, and ecosystems; health, disaster risk management; and water resources, ranging between 3 and 12 (out of 15). Not surprisingly, the health sector obtained the highest scores across all countries given its direct responsibility for health-related activities, as expected. For detailed information on these scores, see Supplementary Tables S4–S15 in the Supplementary Materials.

When analysing other health-impacting sectors, the disaster risk management sector widely addressed several health-related activities across all countries. Specifically, the relationship between health and disaster risk management is usually stated explicitly and is focused on the impact of climate change on healthcare facilities; the burden associated with direct and indirect injuries; human mortality and morbidity, including outbreaks of infectious diseases. For example, the NAPs of Colombia, Paraguay, and Peru specify the impact of El Niño climate events (although El Niño is a climate phenomenon) on housing, infrastructure, disease outbreaks, and household income. Some countries even estimated hazard risks for future projected El Niño events (e.g., Argentina, Chile, and Peru). However, no country explicitly included a health-related institution when discussing national entities that lead planning activities within the disaster risk management sector. Importantly, the disease risk management responsibilities lie within multiple sectors—including the health sector—which creates redundancy in work and communication problems. For example, NAPs for Chile and Paraguay mention that health authorities need to be involved in this sector; however, their specific roles and responsibilities are unclear and not detailed. Additionally, most countries do not mention the funding necessary to strengthen disaster risk management sector and health-related activities, nor if funds were allocated or where the funds would come from. Some countries, such as Chile and Colombia (NAPs), mention that the funding amount is in process of being defined or estimated. Regarding indicators for disaster risk management, few countries mention these.

A similar situation is observed with the water resources sector as is seen in the disaster risk management sector. Baseline information on the link between water resources and population health is extensively recognised in plans across almost all countries, with similar level of detail on adaptation proposals; however, the identification of leading institutions, financing, and indicators is not well-developed. Other health-impacting sectors, such as biodiversity, environment and ecosystems, education and communication, energy, industry, cities and infrastructure, and tourism, barely include a health perspective in their narratives and plans.

### Health-sector-focused analysis

**Fig. 2:**
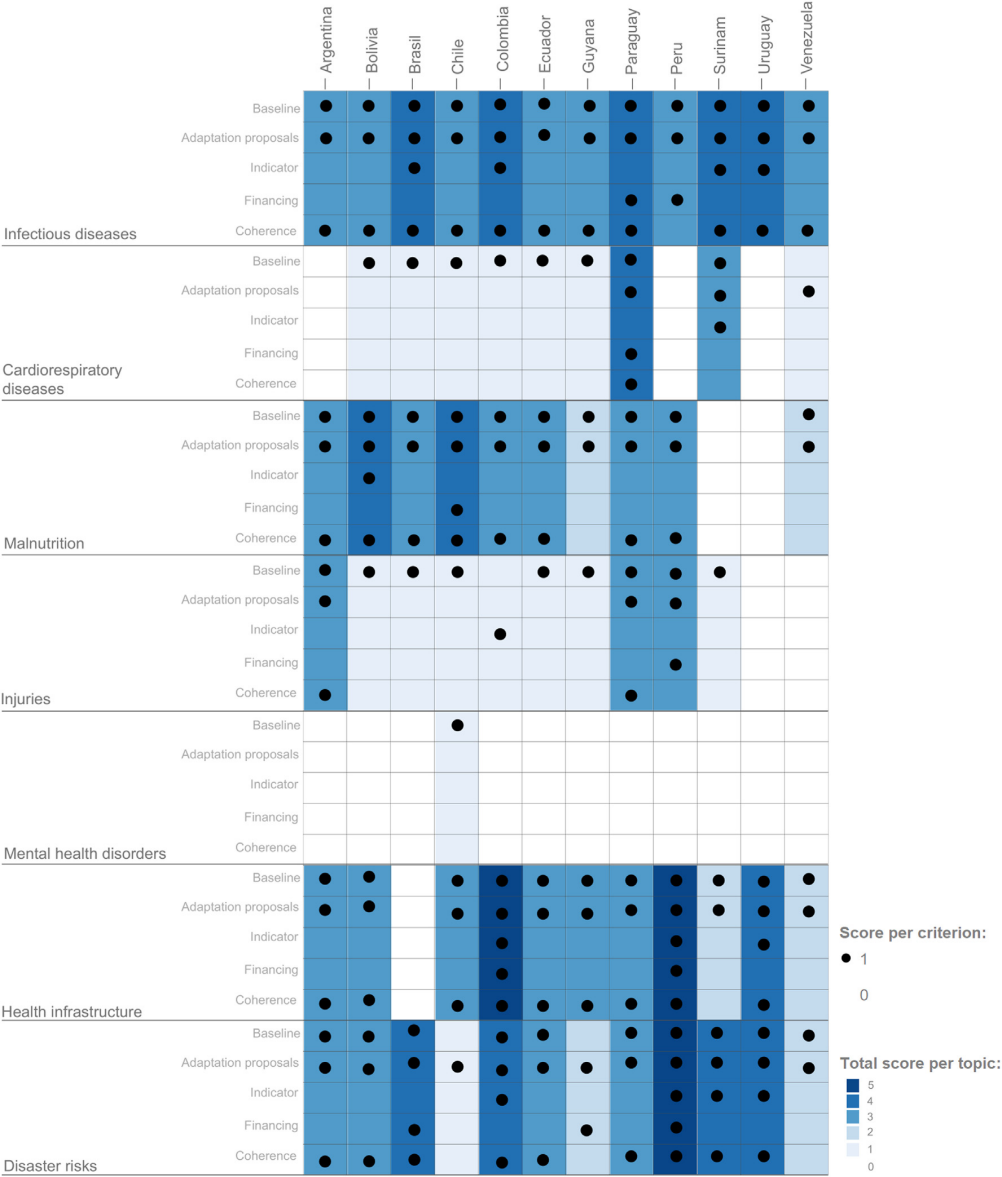
Health-sector-focused analysis of plans in South American countries.

The health-sector-focused analysis showed that all countries recognise that climate change impacts population health, resulting in several health outcomes. As was the case in the intersectoral health analysis, all countries describe and develop adaptation proposals and actions. However, once again, the depth varies according to the climate-sensitive health topic under analysis. Scores related to indicators and funding reveal the lack of information on these criteria. There are wide ranges in details in the plans. For example, Colombia and Peru do include some indicators and funding sources for specific health topics. Fig. 2 summarises the main findings of this analysis, showing individual scores per criterion (black dots) and overall score (blue-scale colour) for each climate-sensitive health topic, for each South American country.

In the health-sector-focused analysis, the highest scores (representing most detail) can be found for health infrastructure, particularly in Colombia and Peru, and disaster risk management in Peru. Other health topics that emerge with detail include infectious diseases and malnutrition. However, there is minimal development on cardiorespiratory diseases, injuries, and mental health disorders, despite these topics being recognized as significant health challenges that are expected to worsen with the impact of climate change (Supplementary Tables S16–S27 in the Supplementary Material). Only Chile includes baseline information on mental health, but does not develop adaptation proposals, indicators, or financing related to it.

Overall, baseline information, adaptation proposals, and coherence for the adaptation proposals scored well, whereas these plans were very limited in detail related to financing and indicators. The coherence criterion evaluated the logicality and consistency between the three criteria: baseline, adaptation proposal, and indicators. Many countries might have not provided clear indicators (see Supplementary Tables S16–S27), but in general they scored well in coherence because there was consistency and logic between the baseline information and the adaptation proposals.

## Discussion

This policy analysis is the first that systematically analyses NAPs, NDCs, and NCs in South America, examining policy documents available up to December 2021. As of that date, seven (out of 12) South American countries have a NAPs and all have NDCs and NCs. However, the in-depth intersectoral health analysis and health-sector-focused analysis revealed that although health is included in these policy documents, the level of detail that is necessary to guide policy makers’ actions is lacking.

For the intersectoral analysis, baseline information and adaptation proposals or plans were the most developed and detailed criteria. However, baseline information was broad and, for the most part, not specific to the vulnerabilities or issues that were specific to that country. Indicators that would be used to evaluate changes over time were available for half of the countries but missing in the rest. There was minimal detail, if any, on the institutions that needed to be involved in this planning and implementation process, and on the funding sources and amounts that would be required. Moreover, our 4-level scoring system for the intersectoral analysis captured differences in how implicit or explicit linkages with health were stated in the national plans. Overall, health topics were mostly covered within the health sector, as would be expected, followed by water resources, disaster risk management, and agriculture and food security sectors. The most implicit or weakest linkages with health were found within the energy, education, and industry sectors.

Overall, this study expands on the global and regional evidence about the limited integration of a health perspective into climatic policies.^60^, ^61^, ^62^ It is important to note that the purpose of this critical analysis is not to assign blame for the identified shortcomings, but rather to assess the progress made thus far and identify opportunities for further strengthening climate plans in the region to ensure proper adaptation to the changing climate.

The intersection of climate change and health is a multifaceted subject. To effectively plan for adaptation to this intricate threat, it is necessary to address its inherent complexity. This can be achieved by integrating multisectoral and multilevel perspectives, employing systems thinking approaches, involving all key stakeholders and entities through the planning and implementation phases, gaining a comprehensive understanding of the current baseline situation at the national and sub-national levels, and detailing the necessary financial mechanisms to implement measures, along with indicators for intra- and inter-country comparisons over time.

At the moment, the findings from this analysis reveal that the planning for most countries is incomplete: the adaptation proposals are not enough. As planning improves, countries must also consider the processes by which these plans will be implemented and should lean on fields such as implementation science^63^^,^^64^ and organizational psychology, to ensure that beyond having a plan, that the processes for their implementation are well thought out and carried out effectively. Fortunately, there are several analyses, guidelines, and toolkits available to support governments in establishing these plans.^65^, ^66^, ^67^ For example, the World Health Organisation (WHO) has published quality criteria guidelines for Health National Adaptation Plans,^68^ helping countries advancing in their sectoral plans. The Operational framework for building climate resilient health systems, also published by the WHO, offers guidance to the health sector on effectively addressing challenges posed by climate variability and change. It targets public health professionals, health managers, and decision-makers in related sectors like nutrition, water and sanitation, and emergency management.^69^

Planning for adaptation related to climate change requires a long-term vision and an intersectoral and interdisciplinary team of individuals who can lead this work overtime. And yet, many countries in South America have political turmoil and instability that makes planning and working on long-term projects difficult. Moreover, unlike countries in Europe or North America, this region is not starting this adaptation planning process with an established strong health system. The COVID-19 pandemic revealed the deficiencies in the current health systems in South America: a fragmented health care system, lack of data to guide decision-making, and one in which many health facilities do not have the basic infrastructure of potable water, electricity or internet for the most basic functions.^70^ This and other epidemics, such as that of dengue, have demonstrated that the systems tend to be reactive and curative instead of being proactive and preventive. Adaptation planning must start with ensuring that the basic health infrastructure is already met, so we can build upon it; in this case, adaptation proposals can be undermined by the lack of basic services, limiting adequate climate adaptation (see summary in Panel 1 below).

*Panel 1* **Filling gaps to advance climate adaptation for health.**

As this paper highlights, there are gaps in national plans related to climate change and health that must be addressed to better safeguard the well-being of South Americans, which include:

• Support national assessments for climate-health vulnerabilities, identifying strengths/weaknesses in policies, resources, and infrastructure.
• Strengthen plans by using baseline assessments to prioritise adaptation actions, define budgets, and identify key institutions and officials for leadership and involvement.
• Within countries, engage different sectors and key stakeholders for holistic solutions to climate-health vulnerabilities. Promote inclusive, cross-sectoral climate adaptation policies involving government and society for sustainability.
• Consider the provision of basic services (i.e., potable water, electricity) as a fundamental step for climate adaptation. Locally assess the distribution of basic services and include this information in the identification of the most vulnerable populations to climate change.
• Increase coherence of national plans by ensuring proposed adaptation actions have associated measurable baseline and evaluation indicators.

This study has limitations. First, despite assigning two independent reviewers to review all country plans and developing a scoring card to score the plans, there is still much subjectiveness in this process, particularly due to the size and extent of these various policy documents. It is possible that, even with two reviewers going through all documents for a country, some information in other related documents was missed. Second, the study used official published documents that were available on the UNFCCC. It is possible that different countries have more detailed plans being used for this planning, and not their NAPs, NDCs, or NCs. Our analysis offers a comprehensive panoramic assessment of progress, as well as weaknesses, that could be used to guide policy discussions regarding climate change adaptation and the improvement of health in the region. However, they might not sufficiently capture the country-level intricacies inherent to this diverse group of nations. The real contribution of this work will be possible if these results can be discussed with key stakeholders at the country level, allowing for a shared understanding of the strengths and weaknesses of their current plans, in order for them to improve the planning and implementation of health-related climate change adaptation measures. While it is our intention that the current analysis is a first comprehensive step, it is also recognized that a more detailed country-level exploration is warranted, considering the intricate nuances in existence within these plans and their alignment with practical efforts on the ground. Third, the research team developed the scoring cards for this analysis. The team met numerous times with different policy experts and researchers as this analytical process was being developed. The team recognised that there were some cross-cutting elements that are important in any plan: the “who, when, where, and what” of the planning. In the initial analysis, the Sustainable Livelihoods^15^ and One Health^16^ approaches were considered for coding and categorising data, but they did not adequately fit the analysis that was required; the team needed a way to evaluate the level of detail and depth regarding various topics and planning components. After multiple starts and stops, as well as discussions with outside experts, the team developed the described scoring cards. A recent publication by the Global Climate Health Alliance (GCHA) analysed NDCs to determine the extent to which governments’ national climate commitments recognise and respond to the abundant linkages with health; this was published after our analytical process had been completed. The GCHA publication examined: integrated governance, health impacts, health sector action, health co-benefits, economics and finance, and monitoring and implementation.^62^ Albeit a different scoring system, the use of a similar approach developed by an entirely different team (GCHA) instils confidence in the appropriateness of the methodology and indicators used in our study for this type of analysis of policies and data. However, the scoring cards developed for this study could be evaluated by a multisectoral and global team, and improved upon and standardised for future use in South America or other regions of the world. Additionally, the GCHA analysis included other topics, such as health co-benefits. Identifying, declaring, and measuring health co-benefits can provide a strong incentive for climate policy,^71^^,^^72^ and relevant and important to examine in the context of South American national plans; these have already been analysed and discussed by another manuscript within this Lancet series. The WHO survey on climate change and health^73^ is a good alternative to this study and provides faster information; however, it relies on self-reports which are likely to be biased and subject to conflicts of interest. Moreover, some South American countries, such as Chile, did not participate in the survey, leaving some relevant gaps. Another challenge of this study, although not a methodological limitation, is related to the fact that NAPs, NDCs, and NCs start with the premises of having secured basic commodities (e.g., potable water, sewage grid, electricity); the reality is that many health facilities throughout the region do not have these basic commodities to begin with.

Health is an essential aspect of human life and a fundamental right recognised by the United Nations.^74^ South American countries, like other countries around the world, have a responsibility to prioritise climate policies that safeguard public health from climate hazards. It is critical to integrate health—and even place health at the centre of—NAPs, NDCs, and NCs, to develop effective strategies for mitigating the impacts of climate change and adapting to changing health risks. Many actions in health-impacting sectors (e.g., energy, industry, cities, agriculture) are aimed at mitigating climate change, without realizing that well implemented mitigation strategies can yield health co-benefits,^71^^,^^75^ such as reducing air pollution and promoting physical activity, thus improving well-being and mental health. The cities and infrastructure sector is particular important in South America where 80% of the population live in cities and most likely climate change would only cause more intense migration towards urban areas. Therefore, building cities that are resilient to climate change is imperative and pressing. By including a health perspective in NAPs, NDCs, and NCs, South American countries can identify and implement policies that promote both climate and health goals, potentiating multiple gains, optimising resources, and advancing in multiples agendas, including the agenda 2030.^76^

The plans put forth by South American countries in response to climate change represent significant efforts to address the impact of climate on health. However, there is still room for improvement in terms of safeguarding public health and reaping the full benefits of climate policies. Ultimately, the goal of these plans, especially National Adaptation Plans, should be to build the resilience of existing societal systems, including health care.

## Contributors

VAPS, AV, KC, ZM, RCN: conceptualization and study design. VAPS, RCN: supervision. AV, KC, AP, AS, CI, ML, VF: investigation and data curation. VAPS, AV, KC, YKPS: formal analysis. VAPS, AV, YKPS, RCN: writing-original draft. VAPS, AV, YKPS, SMH: writing-review.

## Declaration of interests

AV, KC were supported by the Wellcome Trust (209734/Z/17/Z). RCN was funded by K01AI139284 (NIH-NIAID).
