# Supplementary material for: A critical analysis of national plans for climate adaptation for health in South America
Source: Lancet Reg Health Am. 2023 Oct 9;26:100604. doi: 10.1016/j.lana.2023.100604 (PMC10593566; doi:10.1016/j.lana.2023.100604)
Supplement: Supplementary Materials [file mmc1.docx]

**Supplementary Materials**

**Identifying Gaps and Opportunities for Climate Health Adaptation: A Critical Analysis of National Adaptation Plans for Climate Change in South America**

**Table S1.** Plans included by country.

| **Country** | **NAP (year)** | **Sectoral NAP** | **NDC (year)** | **NC (year)** |
| --- | --- | --- | --- | --- |
| Argentina | - | - | Yes (2021) | Yes (2015) |
| Bolivia | - | - | Yes (2022) | Yes (2020) |
| Brazil | Yes (2016) | Yes (n=1)#  - Agriculture  - Biodiversity & Ecosystems  - Cities  - Disaster Risk Management  - Industry & Mining  - Infrastructure  - Vulnerable Populations  - Water resources  - Health  - Food and Nutritional Security  - Coastal Zones | Yes (2022) | Yes (2020) |
| Chile | Yes (2017) | Yes (n=4)  - Health*  - Agriculture  - Biodiversity  - Fisheries & Aquaculture | Yes (2020) | Yes (2021) |
| Colombia | Yes (2018) | - | Yes (2020) | Yes (2017) |
| Ecuador | - | - | Yes (2019) | Yes (2017) |
| Guyana | - | - | Yes (2016) | Yes (2012) |
| Paraguay | Yes (2020) | - | Yes (2021) | Yes (2017) |
| Peru | Yes (2021) | - | Yes (2020) | Yes (2016) |
| Surinam | Yes (2020) | - | Yes (2019) | Yes (2016) |
| Uruguay | Yes (2019/2021)* | Yes (n=3)  - Agriculture  - Cities  - Coastal | Yes (2017) | Yes (2019) |
| Venezuela | - | - | Yes (2021) | Yes (2018) |

Source: UNFCCC portal https://napcentral.org/sectoral-naps

* These documents exist, but they were not available from UNFCCC. The Chilean adaptive plan for the health sector can be found here: https://mma.gob.cl/wp-content/uploads/2018/06/Plan-de-adaptacio%CC%81n-al-cambio-clima%CC%81tico-para-salud_2016.pdf

# Instead of separate sectoral NAPs, Brazil has one document containing 11 sectoral strategies

**Table S2.** Criteria for considering explicit and implicit information related to health.

| **Health-determining sector** | **Explicit information** | **Implicit information** |
| --- | --- | --- |
| Agriculture and food production | Agriculture, livestock, farming, aquaculture or fishing sector when related to health, e.g. food variety. For example, crops and climatic extremes that affect them, as well as direct impacts due to natural disasters such as floods, droughts, frost, sea level rise, landslides, mass movements, etc. The use of pesticides, fertilizers, nutrition and animal health are also considered. Health and relative terms are also considered. | It should contain key words such as: health, diseases and/or relationship between diseases, welfare, mortality and morbidity, safety (food contamination), obesity, anaemia. |
| Biodiversity, environment, and ecosystems | For example, pollution, ocean acidification, deforestation, forest fires, land use change that have a (written) implications on populations, communities or individuals, groups of people, people's well-being, and diseases. Also, consider ecosystem services for the health/well-being of the population. Damage and associated losses or diseases and human life. Also, direct impacts from natural disasters such as floods, sea level rise, landslides, mass movements, etc. | It should contain key words such as: health, diseases and/or relationship between diseases, welfare, mortality and morbidity. Zoonotic diseases. |
| Cities and infrastructure | Cities and infrastructure when related to health. For example, transportation (access to transportation including pedestrian access - in the sense of well-being or health - that it is written) and pollution that affects health (that it is written), housing. Also, zoning, use of public space and land use (industrial, urban, rural), and land use planning concerning people's welfare or health. Also, consider the waste collection, construction of sanitary landfills, sewage networks, and public safety. Also, direct impacts due to natural disasters such as floods, sea level rise, landslides, mass movements, etc. | It should contain key words such as health, diseases, and/or relationship between diseases, well-being, mortality, and morbidity. |
| Disaster risk management | Natural hazards due to climate change. For example, floods, droughts, landslides, mass movements, landslides, alluvium, frost, glacial retreat, alluvium, glacial landslides, etc. | Impacts of natural disasters that have an explicit impact on health, i.e. physical injuries, respiratory infections, etc |

Continue on next page

**Table S2 (cont).** Criteria for considering explicit and implicit information related to health.

| **Health-determining sector** | **Explicit information** | **Implicit information** |
| --- | --- | --- |
| Education and communication | Communication and education when related to health.  For example, the inclusion of health and CC in the curricula of regular basic education and higher education. Also, if they make climate change and health dissemination programs, e.g.: generation of dissemination material (example regarding Covid-19). | It should contain key words such as: Health, diseases and/or relationship between diseases, wellbeing, mortality, and morbidity. |
| Energy | Energy when related to health/wellness. For example, mention of availability and use of renewable and non-renewable energy for use in homes or hospitals (that is written), generation and/or distribution of power for domestic, and hospital use. Also, pollution from the energy that affects the health/welfare of people, communities, etc., as well as clean energy or renewable energy for air quality improvement. | It must contain key words such as: Health, diseases and/or relationship between diseases, well-being, mortality and morbidity. Supply to hospitals, medical posts, institutes  (medical infrastructure). |
| Health | This is what the country considers within the health sector. For example, health workforce, medical services, surveillance systems, early warning systems, vector-borne diseases, water-borne diseases, spoiled food, malnutrition, cardiorespiratory diseases, injuries, morbidity, mortality, mental health impacts. | It should contain keywords such as “health”, “diseases”, “wellbeing”, “mortality”, “morbidity” |
| Industry | When the impacts of climate change, such as extreme weather, natural disasters, etc., are related to the health of workers, or mean an increase in occupational accidents. As well as when the contamination of rivers, oceans, soil and/or air caused by the industry means the affectation on communities, people. | It must contain key words such as: Health, diseases and/or relationship between diseases, well-being, mortality, and morbidity. |
| Tourism | Tourism when related to health. For example, pollution from tourism and its impact on the health/well-being of individuals or the community (that is written). Also consider mental health. Also, direct impacts on the sector due to natural disasters such as floods, sea level rise, landslides, mass movements, etc. | It should contain key words such as: health, diseases and/or relationship between diseases, well-being, mortality, and morbidity. |
| Water resources | For example, the relationship between water and diseases, vector diseases, and contamination. Also, direct impacts of natural disasters such as floods, sea level rise, landslides, mass movements, etc. | It should contain keywords such as “health”, “diseases”, “relationship between diseases”, “well-being”, “mortality” & “morbidity”, “contamination of drinking” “water reservoirs”, “drinking water quality”, “water availability”, “water scarcity”, “sanitation”, “water & drainage”. |

**Table S3.** Criteria for considering climate-related umbrella topics.

| **Topics** | **Definition** |
| --- | --- |
| Infectious diseases | - Diseases transmitted by pathogens, vectors or livestock or domestic animals. - Diseases transmitted by water, food or contaminated environment. - There may also be a mention in the monitoring, surveillance, or early warning system for re/emerging disease outbreaks. |
| Cardiorespiratory diseases | Topics related to environmental pollution, pneumonia, early warning or health personnel and medical care.  Respiratory diseases:   - Associated with infections: Common flu, influenza, pneumonia or similar. - Chronic disorder: Allergies/Asthma, COPD or Chronic Obstructive Pulmonary Disease characterized by marked shortness of breath. - Depending on the component of the respiratory system affected: sinusitis, pharyngitis, tonsillitis, laryngitis, bronchitis or combinations.   Cardiac diseases: hypertension, heart failure/failure/attack/arrest or stroke (altered blood supply to the brain due to damage to the heart, arteries and/or veins). |
| Malnutrition | - Topics related to food security.   Alterations due to nutritional caloric   - - Anaemia (iron deficiency or blood alteration)   - Marasmo (total caloric deficiency) – frequent in children   - Kwashiorkor (protein deficiency) – frequent in children   Alterations due to excessive food consumption   - - Overweight or obesity     - Predisposition to chronic diseases (diabetes, hypertension, cancer, etc) |
| Injuries | Due to a natural hazard or disaster (e.g., flood, tsunami, earthquake, etc.) and/or mention of early warning.   - Injuries due to accidents or situations of violence manifested as trauma (exposed injury), traumatisms (exposed injury), contusions (injury with no skin damage) or loss of physical integrity due to natural hazards and disasters (floods, landslides, tsunamis, earthquakes, or similar), human-induced hazards (fires, acid rains, droughts or similar) addressed with early warning systems. - Morbidity as an increase in preventable diseases associated with climate change preventable by improving of social determinants (education, living conditions, employment, or support networks/programs) and/or environmental (temperature, air, clean water and/or food security). - Mortality as increase in deaths/deaths/deaths associated with or caused by climate change due to natural or human-induced disasters. |
| Mental health | Access and health personnel  **Mental disorders:**   - Anxiety disorder (general anxiety, stress, panic, or phobias). - Altered mood (depression, bipolarity - marked mood swings that affect lifestyle, cyclothymia - mood swings that do not limit activities). - Substance abuse (alcoholism, drug addiction or similar) - Eating disorders (anorexia - voluntary food restriction or bulimia - excessive eating). |

Continue on next page

**Table S3 (cont).** Criteria for considering climate-related umbrella topics.

| **Topics** | **Definition** |
| --- | --- |
| Infrastructure | It refers to the existing basic conditions or that a health centre (hospital, post, health/community centre) must have, such as drinking water, drainage, electricity and communications to provide a health/care service (patient care activities, disease prevention and promotion of healthy lifestyles) appropriate. They are also the basic sanitation conditions in homes or in public places or those that the general population has. Includes WASH: Water and Sanitation/Hygiene  It is also considered access to health, health insurance. It also refers to the number of medical centres, capacity, size, health personnel, health human resources. |
| Disaster risk management | It refers to the reaction to a natural hazard, the surveillance system, early warning, health personnel and medical care.  In addition to the plans, personnel, supplies or services that are available in emergency situations and natural or human-induced disasters associated with climate change to provide a health/care service. |

**Table S4.** Scoring for intersectoral health analysis, Argentina

| **Sector** | **Baseline** | **Adaptation proposals** | **Leading and involvement** | **Financing** | **Indicators** | **Total** |
| --- | --- | --- | --- | --- | --- | --- |
| **Agriculture and food production** | 2 | 1 | 0 | 0 | 0 | 3 |
| **Biodiversity, environment, and ecosystems** | 2 | 2 | 0 | 0 | 0 | 4 |
| **Cities and infrastructure** | 2 | 2 | 0 | 0 | 0 | 4 |
| **Disaster risk management** | 2 | 1 | 0 | 0 | 0 | 3 |
| **Education and communication** | 0 | 0 | 0 | 0 | 0 | 0 |
| **Energy** | 2 | 1 | 0 | 0 | 0 | 3 |
| **Health** | 3 | 2 | 0 | 0 | 0 | 5 |
| **Industry** | 2 | 0 | 0 | 0 | 0 | 2 |
| **Tourism** | 2 | 0 | 0 | 0 | 0 | 2 |
| **Water resources** | 2 | 1 | 0 | 0 | 0 | 3 |

**Table S5.** Scoring for intersectoral health analysis, Bolivia

| **Sector** | **Baseline** | **Adaptation proposals** | **Leading and involvement** | **Financing** | **Indicators** | **Total** |
| --- | --- | --- | --- | --- | --- | --- |
| **Agriculture and food production** | 2 | 3 | 0 | 0 | 3 | 8 |
| **Biodiversity, environment, and ecosystems*** | 3 | 2 | 0 | 0 | 1 | 6 |
| **Cities and infrastructure** | 1 | 1 | 0 | 0 | 0 | 2 |
| **Disaster risk management** | 3 | 2 | 0 | 0 | 0 | 5 |
| **Education and communication** | 0 | 0 | 0 | 0 | 0 | 0 |
| **Energy** | 2 | 3 | 0 | 0 | 1 | 6 |
| **Health** | 3 | 2 | 2 | 0 | 2 | 9 |
| **Industry** | 0 | 0 | 0 | 0 | 0 | 0 |
| **Tourism** | 0 | 0 | 0 | 0 | 0 | 0 |
| **Water resources** | 3 | 3 | 0 | 0 | 3 | 9 |

*It recognises ecosystem as a living organism.

**Table S6.** Scoring for intersectoral health analysis, Brazil

| **Sector** | **Baseline** | **Adaptation proposals** | **Leading and involvement** | **Financing** | **Indicators** | **Total** |
| --- | --- | --- | --- | --- | --- | --- |
| **Agriculture and food production** | 3 | 2 | 0 | 1 | 1 | 7 |
| **Biodiversity, environment, and ecosystems** | 3 | 1 | 0 | 0 | 0 | 4 |
| **Disaster risk management** | 3 | 3 | 1 | 1 | 2 | 10 |
| **Cities and infrastructure** | 3 | 2 | 0 | 0 | 0 | 5 |
| **Education and communication*** | 2 | 2 | 0 | 0 | 2 | 6 |
| **Energy** | 1 | 0 | 0 | 0 | 0 | 1 |
| **Health** | 3 | 3 | 2 | 0 | 2 | 10 |
| **Industry** | 2 | 0 | 0 | 0 | 0 | 2 |
| **Tourism** | 0 | 0 | 0 | 0 | 0 | 0 |
| **Water resources** | 2 | 3 | 0 | 1 | 0 | 6 |

*Cross-cutting sector

**Table S7.** Scoring for intersectoral health analysis, Chile

| **Sector** | **Baseline** | **Adaptation proposals** | **Leading and involvement** | **Financing** | **Indicators** | **Total** |
| --- | --- | --- | --- | --- | --- | --- |
| **Agriculture and food production** | 3 | 2 | 0 | 2 | 0 | 7 |
| **Biodiversity, environment, and ecosystems** | 2 | 2 | 0 | 0 | 0 | 4 |
| **Disaster risk management*** | 2 | 2 | 2 | 0 | 0 | 6 |
| **Cities and infrastructure** | 2 | 1 | 1 | 0 | 0 | 4 |
| **Education and communication** | 1 | 1 | 0 | 0 | 0 | 2 |
| **Energy** | 0 | 0 | 0 | 0 | 0 | 0 |
| **Health** | 3 | 2 | 3 | 0 | 0 | 8 |
| **Industry** | 0 | 0 | 0 | 0 | 0 | 0 |
| **Tourism** | 1 | 0 | 1 | 0 | 0 | 2 |
| **Water resources** | 0 | 2 | 2 | 0 | 0 | 4 |

*Cross-cutting sector

**Table S8.** Scoring for intersectoral health analysis, Colombia

| **Sector** | **Baseline** | **Adaptation proposals** | **Leading and involvement** | **Financing** | **Indicators** | **Total** |
| --- | --- | --- | --- | --- | --- | --- |
| **Agriculture and food production** | 2 | 1 | 0 | 0 | 0 | 3 |
| **Biodiversity, environment, and ecosystems** | 1 | 2 | 0 | 0 | 1 | 4 |
| **Disaster risk management** | 2 | 2 | 0 | 3 | 1 | 8 |
| **Cities and infrastructure** | 2 | 1 | 0 | 2 | 2 | 7 |
| **Education and communication** | 0 | 0 | 0 | 0 | 0 | 0 |
| **Energy** | 0 | 1 | 0 | 0 | 0 | 1 |
| **Health** | 2 | 3 | 1 | 0 | 3 | 9 |
| **Industry** | 1 | 1 | 0 | 0 | 0 | 2 |
| **Tourism** | 1 | 0 | 0 | 0 | 0 | 1 |
| **Water resources** | 1 | 3 | 0 | 2 | 1 | 7 |

**Table S9.** Scoring for intersectoral health analysis, Ecuador

| **Sector** | **Baseline** | **Adaptation proposals** | **Leading and involvement** | **Financing** | **Indicators** | **Total** |
| --- | --- | --- | --- | --- | --- | --- |
| **Agriculture and food production** | 2 | 2 | 0 | 0 | 0 | 4 |
| **Biodiversity, environment, and ecosystems*** | 2 | 1 | 0 | 0 | 0 | 3 |
| **Disaster risk management** | 2 | 1 | 0 | 0 | 0 | 3 |
| **Cities and infrastructure** | 2 | 2 | 0 | 0 | 0 | 4 |
| **Education and communication** | 1 | 1 | 0 | 0 | 0 | 2 |
| **Energy** | 1 | 1 | 0 | 0 | 0 | 2 |
| **Health** | 3 | 2 | 1 | 0 | 0 | 6 |
| **Industry** | 1 | 1 | 0 | 0 | 0 | 2 |
| **Tourism** | 2 | 0 | 0 | 0 | 0 | 2 |
| **Water resources** | 3 | 2 | 0 | 0 | 0 | 5 |

*It recognises ecosystem as a living organism.

**Table S10.** Scoring for intersectoral health analysis, Guyana

| **Sector** | **Baseline** | **Adaptation proposals** | **Leading and involvement** | **Financing** | **Indicators** | **Total** |
| --- | --- | --- | --- | --- | --- | --- |
| **Agriculture and food production** | 3 | 2 | 1 | 0 | 0 | 6 |
| **Biodiversity, environment, and ecosystems** | 2 | 1 | 0 | 0 | 0 | 3 |
| **Disaster risk management*** | 2 | 2 | 1 | 1 | 0 | 6 |
| **Cities and infrastructure** | 2 | 2 | 0 | 2 | 0 | 6 |
| **Education and communication*** | 0 | 2 | 0 | 0 | 0 | 2 |
| **Energy** | 2 | 1 | 0 | 0 | 0 | 3 |
| **Health** | 3 | 2 | 2 | 0 | 0 | 7 |
| **Industry** | 0 | 0 | 0 | 0 | 0 | 0 |
| **Tourism** | 2 | 1 | 0 | 0 | 0 | 3 |
| **Water resources** | 2 | 2 | 1 | 0 | 0 | 5 |

*Cross-cutting sector

**Table S11.** Scoring for intersectoral health analysis, Paraguay

| **Sector** | **Baseline** | **Adaptation proposals** | **Leading and involvement** | **Financing** | **Indicators** | **Total** |
| --- | --- | --- | --- | --- | --- | --- |
| **Agriculture and food production** | 1 | 1 | 2 | 0 | 0 | 4 |
| **Biodiversity, environment, and ecosystems** | 0 | 2 | 0 | 0 | 0 | 2 |
| **Disaster risk management** | 3 | 3 | 2 | 0 | 0 | 8 |
| **Cities and infrastructure** | 2 | 2 | 0 | 0 | 0 | 4 |
| **Education and communication** | 0 | 1 | 0 | 0 | 0 | 1 |
| **Energy** | 2 | 2 | 0 | 0 | 0 | 4 |
| **Health** | 3 | 2 | 1 | 2 | 0 | 8 |
| **Industry** | 0 | 0 | 0 | 0 | 0 | 0 |
| **Tourism** | 0 | 0 | 0 | 0 | 0 | 0 |
| **Water resources** | 2 | 2 | 1 | 0 | 0 | 5 |

**Table S12.** Scoring for intersectoral health analysis, Peru

| **Sector** | **Baseline** | **Adaptation proposals** | **Leading and involvement** | **Financing** | **Indicators** | **Total** |
| --- | --- | --- | --- | --- | --- | --- |
| **Agriculture and food production** | 3 | 2 | 0 | 2 | 1 | 8 |
| **Biodiversity, environment, and ecosystems** | 2 | 2 | 1 | 2 | 1 | 8 |
| **Disaster risk management*** | 2 | 2 | 0 | 0 | 2 | 6 |
| **Cities and infrastructure** | 1 | 0 | 0 | 0 | 0 | 1 |
| **Education and communication** | 1 | 1 | 0 | 0 | 2 | 4 |
| **Energy** | 0 | 0 | 0 | 0 | 0 | 0 |
| **Health** | 3 | 2 | 2 | 2 | 3 | 12 |
| **Industry** | 0 | 0 | 0 | 0 | 0 | 0 |
| **Tourism** | 1 | 0 | 0 | 0 | 0 | 1 |
| **Water resources** | 3 | 2 | 1 | 2 | 2 | 10 |

*Cross-cutting sector

**Table S13.** Scoring for intersectoral health analysis, Suriname

| **Sector** | **Baseline** | **Adaptation proposals** | **Leading and involvement** | **Financing** | **Indicators** | **Total** |
| --- | --- | --- | --- | --- | --- | --- |
| **Agriculture and food production** | 2 | 2 | 0 | 1 | 1 | 6 |
| **Biodiversity, environment, and ecosystems** | 2 | 2 | 0 | 2 | 1 | 6 |
| **Disaster risk management*** | 2 | 1 | 0 | 0 | 1 | 4 |
| **Cities and infrastructure** | 2 | 2 | 0 | 0 | 0 | 4 |
| **Education and communication** | 1 | 2 | 0 | 0 | 1 | 4 |
| **Energy** | 2 | 2 | 0 | 1 | 1 | 6 |
| **Health** | 3 | 3 | 0 | 0 | 2 | 8 |
| **Industry** | 1 | 2 | 0 | 1 | 1 | 5 |
| **Tourism** | 1 | 1 | 0 | 0 | 0 | 2 |
| **Water resources** | 3 | 2 | 0 | 0 | 2 | 7 |

*Cross-cutting sector

**Table S14.** Scoring for intersectoral health analysis, Uruguay

| **Sector** | **Baseline** | **Adaptation proposals** | **Leading and involvement** | **Financing** | **Indicators** | **Total** |
| --- | --- | --- | --- | --- | --- | --- |
| **Agriculture and food production** | 2 | 2 | 1 | 2 | 2 | 9 |
| **Biodiversity, environment, and ecosystems** | 0 | 0 | 0 | 0 | 0 | 0 |
| **Disaster risk management** | 2 | 2 | 0 | 0 | 1 | 5 |
| **Cities and infrastructure** | 1 | 2 | 2 | 0 | 2 | 7 |
| **Education and communication** | 1 | 2 | 0 | 0 | 1 | 4 |
| **Energy** | 1 | 1 | 0 | 0 | 0 | 2 |
| **Health** | 2 | 2 | 2 | 0 | 3 | 9 |
| **Industry** | 0 | 0 | 0 | 0 | 0 | 0 |
| **Tourism** | 1 | 1 | 0 | 0 | 0 | 2 |
| **Water resources** | 1 | 1 | 0 | 0 | 1 | 3 |

**Table S15**. Scoring for intersectoral health analysis, Venezuela

| **Sector** | **Baseline** | **Adaptation proposals** | **Leading and involvement** | **Financing** | **Indicators** | **Total** |
| --- | --- | --- | --- | --- | --- | --- |
| **Agriculture and food production** | 1 | 3 | 0 | 0 | 0 | 4 |
| **Biodiversity, environment, and ecosystems** | 0 | 0 | 0 | 0 | 0 | 0 |
| **Disaster risk management** | 1 | 2 | 0 | 0 | 1 | 4 |
| **Cities and infrastructure** | 1 | 2 | 0 | 0 | 2 | 5 |
| **Education and communication** | 1 | 2 | 0 | 0 | 0 | 3 |
| **Energy** | 0 | 2 | 0 | 0 | 0 | 2 |
| **Health** | 2 | 3 | 1 | 0 | 0 | 6 |
| **Industry** | 1 | 2 | 0 | 0 | 1 | 4 |
| **Tourism** | 0 | 0 | 0 | 0 | 0 | 0 |
| **Water resources** | 1 | 3 | 0 | 0 | 2 | 6 |

**Table S16.** Scoring for health-focused analysis, Argentina

| **Topic** | **Baseline** | **Adaptation proposals** | **Indicators** | **Financing** | **Coherence** | **Total** |
| --- | --- | --- | --- | --- | --- | --- |
| **Infectious diseases** | X | X |  |  | X | 3 |
| **Cardiorespiratory diseases** |  |  |  |  |  | 0 |
| **Malnutrition** | X | X |  |  | X | 3 |
| **Injuries** | X | X |  |  | X | 3 |
| **Mental health** |  |  |  |  |  | 0 |
| **Infrastructure** | X | X |  |  | X | 3 |
| **Disaster risk management** | X | X |  |  | X | 3 |

**Table S17.** Scoring for health-focused analysis, Bolivia

| **Topic** | **Baseline** | **Adaptation proposals** | **Indicators** | **Financing** | **Coherence** | **Total** |
| --- | --- | --- | --- | --- | --- | --- |
| **Infectious diseases** | X | X |  |  | X | 3 |
| **Cardiorespiratory diseases** | X |  |  |  |  | 1 |
| **Malnutrition** | X | X | X |  | X | 4 |
| **Injuries** | X |  |  |  |  | 1 |
| **Mental health** |  |  |  |  |  | 0 |
| **Infrastructure** | X* | X* |  |  | X | 3 |
| **Disaster risk management** | X | X |  |  | X | 3 |

*Associated with energy/electricity

**Table S18.** Scoring for health-focused analysis, Brazil

| **Topic** | **Baseline** | **Adaptation proposals** | **Indicators** | **Financing** | **Coherence** | **Total** |
| --- | --- | --- | --- | --- | --- | --- |
| **Infectious diseases** | X | X | X |  | X | 4 |
| **Cardiorespiratory diseases** | X |  |  |  |  | 1 |
| **Malnutrition** | X | X |  |  | X | 3 |
| **Injuries** | X |  |  |  |  | 1 |
| **Mental health** |  |  |  |  |  | 0 |
| **Infrastructure** |  |  |  |  |  | 0 |
| **Disaster risk management** | X | X |  | X | X | 4 |

**Table S19.** Scoring for health-focused analysis, Chile

| **Topic** | **Baseline** | **Adaptation proposals** | **Indicators** | **Financing** | **Coherence** | **Total** |
| --- | --- | --- | --- | --- | --- | --- |
| **Infectious diseases** | X | X | X |  | X | 4 |
| **Cardiorespiratory diseases** | X |  |  |  |  | 1 |
| **Malnutrition** | X | X |  | X | X | 4 |
| **Injuries** | X |  |  |  |  | 1 |
| **Mental health** | X |  |  |  |  | 1 |
| **Infrastructure** | X | X |  |  | X | 3 |
| **Disaster risk management** |  | X |  |  |  | 1 |

**Table S20.** Scoring for health-focused analysis, Colombia

| **Topic** | **Baseline** | **Adaptation proposals** | **Indicators** | **Financing** | **Coherence** | **Total** |
| --- | --- | --- | --- | --- | --- | --- |
| **Infectious diseases** | X | X | X |  | X | 4 |
| **Cardiorespiratory diseases** | X |  |  |  |  | 1 |
| **Malnutrition** | X | X |  |  | X | 3 |
| **Injuries** |  |  | X |  |  | 1 |
| **Mental health** |  |  |  |  |  | 0 |
| **Infrastructure** | X | X | X | X | X | 5 |
| **Disaster risk management** | X | X | X |  | X | 4 |

**Table S21.** Scoring for health-focused analysis, Ecuador

| **Topic** | **Baseline** | **Adaptation proposals** | **Indicators** | **Financing** | **Coherence** | **Total** |
| --- | --- | --- | --- | --- | --- | --- |
| **Infectious diseases** | X | X |  |  | X | 3 |
| **Cardiorespiratory diseases** | X |  |  |  |  | 1 |
| **Malnutrition** | X | X |  |  | X | 3 |
| **Injuries** | X |  |  |  |  | 1 |
| **Mental health** |  |  |  |  |  | 0 |
| **Infrastructure** | X | X |  |  | X | 3 |
| **Disaster risk management** | X | X |  |  | X | 3 |

**Table S22.** Scoring for health-focused analysis, Guyana

| **Topic** | **Baseline** | **Adaptation proposals** | **Indicators** | **Financing** | **Coherence** | **Total** |
| --- | --- | --- | --- | --- | --- | --- |
| **Infectious diseases** | X | X |  |  | X | 3 |
| **Cardiorespiratory diseases** | X |  |  |  |  | 1 |
| **Malnutrition** | X | X |  |  |  | 2 |
| **Injuries** | X |  |  |  |  | 1 |
| **Mental health** |  |  |  |  |  | 0 |
| **Infrastructure** | X | X |  |  | X | 3 |
| **Disaster risk management** |  | X |  | X |  | 2 |

**Table S23.** Scoring for health-focused analysis, Paraguay

| **Topic** | **Baseline** | **Adaptation proposals** | **Indicators** | **Financing** | **Coherence** | **Total** |
| --- | --- | --- | --- | --- | --- | --- |
| **Infectious diseases** | X | X |  | X* | X | 4 |
| **Cardiorespiratory diseases** | X | X |  | X** | X | 4 |
| **Malnutrition** | X | X |  |  | X | 3 |
| **Injuries** | X | X |  |  | X | 3 |
| **Mental health** |  |  |  |  |  | 0 |
| **Infrastructure** | X | X |  |  | X | 3 |
| **Disaster risk management** | X | X |  |  | X | 3 |

*Global and specific amount: For food-borne diseases: USD 21.3 million. Dengue: USD 16.4 million; **Specific amount for Acute Respiratory Infections: USD 42.5 million.

**Table S24.** Scoring for health-focused analysis, Peru

| **Topic** | **Baseline** | **Adaptation proposals** | **Indicators** | **Financing** | **Coherence** | **Total** |
| --- | --- | --- | --- | --- | --- | --- |
| **Infectious diseases** | X | X |  | X |  | 3 |
| **Cardiorespiratory diseases** |  |  |  |  |  | 0 |
| **Malnutrition** | X | X |  |  | X | 3 |
| **Injuries** | X | X |  | X |  | 3 |
| **Mental health** |  |  |  |  |  | 0 |
| **Infrastructure** | X | X | X | X | X | 5 |
| **Disaster risk management** | X | X | X | X | X | 5 |

**Table S25.** Scoring for health-focused analysis, Suriname

| **Topic** | **Baseline** | **Adaptation proposals** | **Indicators** | **Financing** | **Coherence** | **Total** |
| --- | --- | --- | --- | --- | --- | --- |
| **Infectious diseases** | X | X | X |  | X | 4 |
| **Cardiorespiratory diseases** | X | X | X |  |  | 3 |
| **Malnutrition** |  |  |  |  |  | 0 |
| **Injuries** | X |  |  |  |  | 1 |
| **Mental health** |  |  |  |  |  | 0 |
| **Infrastructure** | X | X |  |  |  | 2 |
| **Disaster risk management** | X | X | X |  | X | 4 |

**Table S26.** Scoring for health-focused analysis, Uruguay

| **Topic** | **Baseline** | **Adaptation proposals** | **Indicators** | **Financing** | **Coherence** | **Total** |
| --- | --- | --- | --- | --- | --- | --- |
| **Infectious diseases** | X | X | X |  | X | 4 |
| **Cardiorespiratory diseases** |  |  |  |  |  | 0 |
| **Malnutrition** |  |  |  |  |  | 0 |
| **Injuries** |  |  |  |  |  | 0 |
| **Mental health** |  |  |  |  |  | 0 |
| **Infrastructure** | X | X | X |  | X | 4 |
| **Disaster risk management** | X | X | X |  | X | 4 |

**Table S27.** Scoring for health-focused analysis, Venezuela

| **Topic** | **Baseline** | **Adaptation proposals** | **Indicators** | **Financing** | **Coherence** | **Total** |
| --- | --- | --- | --- | --- | --- | --- |
| **Infectious diseases** | X | X |  |  | X | 3 |
| **Cardiorespiratory diseases** |  | X |  |  |  | 1 |
| **Malnutrition** | X | X |  |  |  | 2 |
| **Injuries** |  |  |  |  |  | 0 |
| **Mental health** |  |  |  |  |  | 0 |
| **Infrastructure** | X | X |  |  |  | 2 |
| **Disaster risk management** | X | X |  |  |  | 2 |

| **Supplementary Panel S1. Exemplars of detailed information from policies for different criteria**  1. Extract of Chile’s national plans where there is clear designation of “leading institution and involvement”: The policy establishes:  “Title of adaptation measure N°6: Development of a study for the establishment of predictive models of the behaviour of vector diseases and zoonoses associated with climate change.  predictive models of the behaviour of vector-borne diseases and zoonoses associated with climate change. Office in charge of execution: Offices of Zoonoses and Vectors. Collaborative partners: Academia, Ministry of Environment, Public Health Institute, Meteorological Direction, and Livestock and Agriculture Service.” From this extract, the country clearly established the Sector or Ministerial offices in charge of execution.  2. Extract of Colombia’s national plans where there is clear detail of “indicators”: The policy details the indicator related to the percentage of departments (or other territorial units) that implement adaptation actions as per “(number of departmental, district and municipal territorial entities category 1, 2 and 3 that implement actions to adapt to climate change through health promotion and prevention of climate-sensitive diseases. / total number of departmental, district and municipal territorial entities category 1, 2 and 3)*100.”  3. Extract of Paraguay’s national plans where there is clear detail of “financing”:  “The detailed analysis indicates that ARIs will require the largest incremental financial flow, USD 42.5 million, followed by the ADDs, with investments of USD 21.3 million, and then dengue (USD 16.4 million). In total, the flows of both sectors (agriculture and public health) related to adaptation add up to USD 198.6 million constant and discounted to 2005, of additional resources that will be necessary to address climate change in the period 2010-2030, in an average of USD 10 million additional annual investment for the period analysed in both sectors (agriculture and public health)” From this extract, the country clearly identifies and establishes budgets needed according to climate change impacts. |
| --- |
